# Manitoba, the Realm of Veterinarians

**Published:** 1898-07

**Authors:** 


### MANITOBA, THE REALM OF VETERINARIANS.

WHAT can be done by and through organization has been more forcibly demonstrated in Manitoba than in any State or Province of North America. By a union of forces and the personal sacrifices of some of her veterinarians, Manitoba has felt all along governmental lines the influence and true interest of the best citizenship through representatives of the veterinary profession. Alike her people have appreciated how far-reaching are the benefits to be derived by a generous recognition of the profession, and the Province and her chief cities have to-day broad and wise legislation to guard the welfare and interests of all her people in the proper supervision of her food-supply, especially that of meat and milk. Her dairies are carefully guarded and her abattoirs under proper supervision, and, as a result, both producer and consumer are benefited in the largest measure.

Protective legislation has been given the profession, and recent amendments were made more appreciative of higher education, and better things are exacted and demanded by those who are to enter the Province in the future as veterinarians.

All of these matters and many others are directed by the veterinary association, which acts as a central head, directing legislation, conducting all examinations of candidates desirous of practising in the Province, protecting in litigation all her members and prosecuting all offenders. Every qualified member of the profession in the Province becomes a member of the association. WELL DONE, MANITOBA. Others should know more of your achievements.

THE JOURNAL is very glad to be privileged to announce to its readers that a report of the Congress on Tuberculosis, at Paris, will be given in an early number. Dr. Leonard Pearson, delegate from Pennsylvania, has kindly consented to prepare a special report for the JOURNAL, and we hope to thus keep all our readers and supporters fully conversant with the latest thoughts and most recent advances in this very important line of work.

---

A THOUSAND active veterinarians in Canada, yet a layman was recently chosen to teach farmers and stockowners "how to test animals for tuberculosis." Aside from the danger such a movement created, what about her recognition of this vast body of earnest men educated in Canadian colleges, partly by governmental assistance. Are her executive authorities trying to turn the hands of the clock of time backward? Is not this vast body of veterinary sanitary police to be your greatest safeguard from the dangers lurking in your food-supply? Why will you choose to ignore that which they can best give, and which to your people is a right, and why step on such dangerous ground both physically and commercially?

---

EVERY reader of this number of the JOURNAL fully appreciates the value and justice of recognition, with rank, of the veterinarians of the American army. Canada already has gained this for the profession in the mounted service of that country, and fully realizes the disadvantages of the members of the profession in the American army. All points of vantage and all statistics will be valuable to those who are pressing the movement in this country, and our colleagues across the line can help us in this way. It is now the opportune time; it is now that we must force the issue, and it can only be done by united effort. Move the good cause along in your district. It may be by a letter to your Congressman; it may be by an interview; it may be by a few lines in your local paper. Use every honorable means for its achievement.

